# A human-centered designed outreach strategy for a youth contraception navigator program

**DOI:** 10.1016/j.pecinn.2022.100093

**Published:** 2022-10-18

**Authors:** Tracey A. Wilkinson, Bridget Hawryluk, Courtney Moore, Jeffrey F. Peipert, Aaron E. Carroll, Sarah Wiehe, J. Dennis Fortenberry

**Affiliations:** a Indiana University School of Medicine, Department of Pediatrics/Children’s Health Services Research, 410 West 10^th^ Street, HS 2000, Indianapolis, IN 46202, United States of America; b Indiana Clinical Translational Institute, Research Jam, 410 West 10^th^ Street, HS 2000, Indianapolis, IN 46202, United States of America; c Indiana University School of Medicine, Department of Obstetrics and Gynecology, UH 2440, Indianapolis, IN 46202, United States of America; d Indiana University School of Medicine, Department of Pediatrics/Center for Pediatric and Adolescent Comparative and Effective Research, 410 West 10^th^ Street, HS 2000A, Indianapolis, IN 46202, United States of America; e Indiana University School of Medicine, Department of Pediatrics, Division of Adolescent Medicine, 410 West 10^th^ Street, HS 1000, Indianapolis, IN 46202, United States of America

**Keywords:** Adolescents, Contraception, Adolescent Health Services, Access to Health Care, Human-Centered Design, Patient Engagement, Pregnancy, Barriers

## Abstract

**Objective:**

To identify key elements of an outreach strategy for a youth contraception navigator program designed to help young people overcome barriers to contraception access.

**Methods:**

A human-centered design approach was used to engage adolescents aged 15–17 in co-design sessions. Human-centered design techniques, such as affinity diagramming and model building were used to inform key elements of the communication model and the final outreach strategy messages.

**Results:**

Messages focused on the individual, normalizing talking about birth control, acknowledging the challenges to obtaining birth control, explaining how the navigator program works resonate with young people. Having images of diverse participants, offering information about birth control, and showing images of reputable sources will enhance trust.

**Conclusions:**

A name (IN Control) and key elements of an outreach strategy were determined for the navigator program. It is important to work with key stakeholders and co-design the optimal strategy and messages to assure that the intended audience is reached, and the desired behavior change is achieved.

**Innovation:**

Human-centered design techniques can be used to provide insight into programmatic outreach strategies for a contraception navigator program to increase their impact and ultimate success.

## 1. Introduction

Increased contraception access for all people and, in particular, young people reduce the unintended pregnancy rate and downstream effects on health and well-being [1,2]. Approaches to increase access focus on clinical processes that remove economic and logistic barriers for patients as well as increase clinician’s knowledge and technical skills [3–6]. Additional efforts focus on diversifying points of entry into clinical care, such as school-based health centers, digital interventions within clinical settings, mobile health units and utilizing peer health educators [7–10]. These efforts to improve access are critical but tend not to address the holistic set of barriers each young person faces throughout contraception navigation, access, and use.

Young people often lack adequate support for overcoming barriers to obtaining contraception prior to, within and after interactions with clinical care. The current landscape of contraception care and the information that adolescents receive within schools can lack comprehensiveness or may be insufficiently evidenced based [11,12]. A human-centered design (HCD) approach was used to create a navigator program to help young people in the state of Indiana overcome patient-specific barriers to contraception access [13]. The goal of the program is to provide support to overcome individual barriers that exist before, during and after contraception is obtained and for the support to be tailored to be particularly sensitive to youth-specific concerns.

HCD, which is increasingly being used within healthcare, is an iterative design process where stakeholders most closely affected by the solution are engaged in developing the solution [14–19]. This is done by understanding stakeholder “needs, desires and experiences which often transcends that which the people themselves actually realized.” The approach enables stakeholders’ ideas for strategy and direction—rather than those of the academic team—to be brought to life. Without stakeholders’ input, interventions may not resonate with the intended participants or may propagate mistrust and stigma [20,21].

Using a similar HCD approach, we sought to develop an outreach strategy to activate adolescents who desire contraception to seek help through the contraception navigator program. We hypothesized that an HCD approach could be used to identify key elements of the communication model and inform an outreach strategy for the contraception navigator program.

## 2. Methods

### 2.1. Study design and study population

Research Jam (RJ), Indiana Clinical and Translational Sciences Institute’s Patient Engagement Core, provided expertise in human-centered design (HCD) techniques. The HCD approach was used to uncover elements of the Reasoned Action Approach (RAA) that impact intentions and ultimate behavior—in particular normative, behavior and control beliefs around obtaining contraception that can be used in messaging strategies [22,23].

**Fig. 1.**
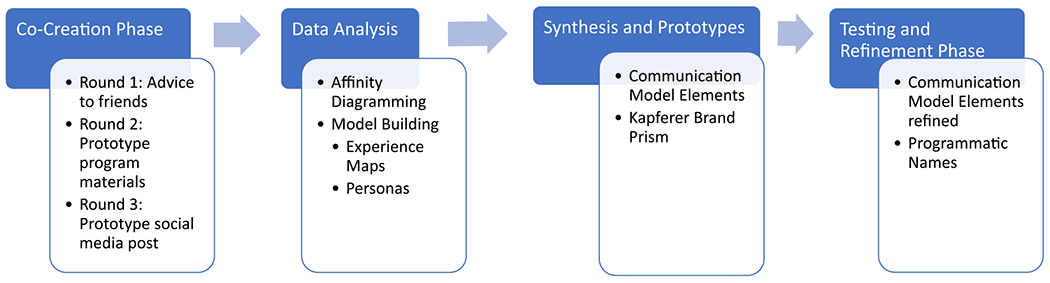
Process overview.

For this project, the objective was to work with youth to co-create messages for a program focused on contraception navigation and access at various parts of a clinical encounter. To meet this objective, the project consisted of two phases: (1) a co-creation phase in which adolescents were asked to begin the message-building process by creating prototypes and (2) a testing and refinement phase in which refined messages created by the team were brought to adolescents for feedback and further refinement (Fig. 1). These processes were used iteratively, and where disagreement arose among research team members, a consensus was ultimately reached through additional discussion.

Adolescent participants were recruited through social media advertisements and relationships with community organizations that interact with young people, such as schools and standing youth advisory boards. The social media advertisements were displayed on Facebook (Appendix A) and directed to young people that met the geographic and age demographics for the study. After clicking on a social media advertisement or the QR code on a flyer, potential participants were directed to an online survey to confirm their interest and eligibility and provide their contact information. Recruitment continued until adequate numbers of participants in each group had been confirmed eligible and had been contacted.

At the time of enrollment, participants were asked to self-identify as White, African American, or Hispanic, and the sessions were conducted separately for each group. We asked participants to choose a group that most reflected their identity, acknowledging not everyone would fit into one alone or at all. Separate design sessions were conducted for each self-identified group to collect data of their specific outreach needs in the event there were differences. Inclusion criteria were the following: age 15–17, ability to speak/read English, access to the internet to utilize the online platform, living in central Indiana, and self-identified as female. The Indiana University Institutional Review Board approved of this study with a waiver of parental permission, participants signed an online written consent. Participants were compensated $75 for the asynchronous prototype message development, and $40 for the one-hour refinement sessions.

### 2.2. Co-creation data collection

For the co-creation phase, the RJ team designed activities to engage adolescents in creating prototype visual and written messages about contraception. Activities took place online using Google Sites, Docs, and Slides and consisted of an ice breaker, three prototyping activities and an opportunity for questions to be asked and answered between both participants and facilitators. At the time of consent, a baseline demographic survey was completed by participants, but no question was required to be answered. Participants were given approximately one week to complete the activities with reminders provided via email. Throughout, participants were encouraged to leave comments about what other participants had contributed to create a conversation and build upon ideas.

Each human-centered design session followed the same agenda, and the activities were released in sequence and built upon one another with various scenario prompts (Table 1). For example, Round 1 asked participants to give advice to three friends who each were facing a different challenge around contraception; Round 2 asked for participants to prototype something for their friend using materials they created or found online and, after introducing the Youth Contraception Navigator (YCN) Program; Round 3 asked participants to prototype a social media post for the YCN program and propose a name for the program.

### 2.3. Analysis

The collected data were downloaded and placed into an online whiteboard called Miro [24]. All text was split so that each discrete statement was on its own digital post-it note, which were color-coded by population. Four human-centered design researchers then reviewed the data to ensure all statements from the original submissions were represented on post-its and reviewed the images submitted by the participants to look for what was included (visuals and text). These descriptions were also added to color-coded post-it notes. The team then began affinity diagramming, a method to discover relationships between ideas and create thematic categories [25]. The initial affinity diagramming was done separately for each of the three race/ethnicity groups to determine whether different populations presented different outreach needs for the navigator program.

After the three populations had themes, these were cross-referenced to see similarities and contrasts between populations. After determining that each population had similar themes for their outreach needs, the themes were merged and analysis continued with model building (Appendix B). Model building allows team members to externalize their thinking about the relationships between the themes. In this way, team members can discuss, refine, and build on each other’s models to create a collective view of the relationships evident in the data. These can be organic models created by team members from scratch or they can utilize existing visual models. For this project, the team used two existing models that fit the goals of the project well: *experience maps* and *personas* [26,27].

Experience mapping examines the data by looking at sequential relationships between the groups of themes (Appendix C). This enabled the RJ team to explore the experience an adolescent would have with the program and identify potential messages that would be appropriate to receive at different points of accessing clinical care. Personas are archetypical visual and written summaries representing a person for which a product or service is being designed and provides a potential user for the HCD team to have in mind during analysis. These fictional adolescents represented different needs and attitudes identified in the data that the team needed to consider when creating messages for the program (Appendix D).

### 2.4. Synthesis

The RJ team then began the synthesis phase which aims to build on the data collected to generate new ideas on “what could be” [25]. In this phase, the team generated elements of the draft communication strategy for the program. To clarify the components of communication for the navigator program, the team utilized a modified version of the Shannon-Weaver communication model [20], which breaks communication into components such as the sender, receiver, message, channel (or medium), and noise (or environment). The developed communication model shows insights related to program messages and opportunities to utilize these messages in program communication. To identify the appropriate personality of the navigator program brand (the sender), the team used the Kapferer brand prism [28,29]. Prototype program messages were then created based on this communication model and brand prism.

### 2.5. Testing and refinement phase data collection

One-hour virtual meetings, also split by race/ethnicity, were conducted to gather feedback on prototype program messages and potential program names. For each of the prototype messages, participants were asked what did and did not resonate and how the message might be improved.

Participants were also asked what elements of representation were important, what words to use and those to avoid, as well as language to identify program navigators and clients to refine the elements of the communication model. Finally, proposed programmatic names were presented and impressions and feedback were solicited.

### 2.6. Analysis

For final communication model elements, patterns in feedback were examined with data from all populations combined. Key elements of the communication model were finalized and examples of how they could be implemented within different settings were proposed by the RJ team. Patterns were also found in the feedback about proposed names and taglines for the YCN Program which helped refine the name ultimately proposed.

## 3. Results

### 3.1. Participation

Six human-centered design research sessions were conducted with adolescents aged 15-17 yrs—three asynchronous sessions (*n* = 19) and three synchronous sessions (*n* = 11). Analysis of affinity mapping themes showed common themes across the three race/ethnicity. Therefore data was combined prior to model building and the development of key insights.

Half of participants were age 16 yrs. Participants self-identified into the following racial/ethnic groups: African American (30%), Hispanic (26.7%) and White (43.3%). A slight majority (53.3%) reported being attracted to boys and 40% were sexually active. A majority (66.7%) agreed or strongly agreed that they knew how to obtain birth control or condoms.

### 3.2. Communication model elements

Key elements of the Communication Model (sender, receiver, message, media, environment) were determined from this analysis and resulted in insights and opportunities for an outreach strategy (Table 2) [30]. Following this data analysis, prototype messages were created (Table 3) that utilized these key insights and elements of the communication model.

When these prototype messages (Table 4) were tested and discussed within a virtual group setting to determine what resonated and what did not, participants provided feedback such as:

“I think it’s a good idea to normalize talking about birth control”.

“That makes me feel more comfortable to ask questions. It also makes me feel safer with decisions about getting birth control.”

“That is a lot of information at once”.

The following was noted by participants when asked who should be visually represented in these messages:

“I think we should represent more minorities in birth control. In black communities and other cultures.”

“Anybody with a uterus because the people who use birth control is a diverse group.”

There was also feedback of using simple words and actual terms, instead of innuendos.

“Plainly please! Don’t sugar coat it, it makes it seem like it’s not very serious.”

“If you sugar coat it, some people won’t understand.”

“Don’t try to be ‘hip’, speak to us normally.”

The overall feedback on messages about the program resulted in the following message guidelines to be considered and final proposed messages (Table 4).

### 3.3. Program naming

Various program names and versions were presented to the participants and included the following: Birth Control Match, Birth Control Shippers, Mission: Control, IN Control, and BC AF (Birth Control Advisor Force).

Participants’ feedback was that the BC AF name was “funny, but maybe not the most professional” and “trying way too hard to be hip, no offense.” Opinions were split on Birth Control Match and Birth Control Shippers with some noting that it was “straight to the point” but others saying it sounds “very generic” and “misleading” because we aren’t shipping birth control directly to participants. The program name “IN Control” resonated the most with participants’ sentiments about the program and additional feedback included that the name was empowering and preserved privacy about the topic because it didn’t mention birth control.

“It’s catchy and it makes the person know/feel that they are in control.”

“I like that it is lighthearted but remains professional.”

“I like this one, it makes me feel powerful.”

“Makes the user feel protected, safe”.

The name also included the abbreviation for the state of Indiana, which was noted by participants to be an additional benefit to let young people within our state aware this program was ours and to further increase trustworthiness.

## 4. Discussion and conclusion

### 4.1. Discussion

A human-centered design approach enabled co-creation of key program communication elements and final messages with young people. Key elements of an outreach strategy, including the name “IN Control” for a youth contraception program were determined. Our findings highlight that adolescents want messages that communicate key elements of the program in a way that normalizes birth control use, helps them feel comfortable learning about birth control options, and acknowledges the challenges to obtaining birth control. The messages need to be focused on the individual and show that the program is trustworthy, open and inviting, and explains how the program itself works. Furthermore, messages should emphasize the focus on client-led decisions and the programmatic support for any decision made as young people may be building competence as health care consumers for the first time.

We examined public health campaigns from government entities and organizations around birth control use for similarities and discovered analogous findings [31]. Namely, having personalized statements or quotes were often used as an effective way of attracting and engaging audiences. Given the personal nature of contraception use, it isn’t surprising that this characteristic is important to incorporate.

Additional research has shown that with online media outreach, attention to how messages are constructed is important when thinking about the audience’s race and ethnicity [32]. It is well known that normative and salient beliefs around contraception vary by race/ethnicity [33–39]. For these reasons, our study design collected data separately in groups based on race and ethnicity. However, during analysis, similar themes were discovered and ultimately resulted in a unified outreach strategy for the “IN Control” navigator program. Given the sample size of this study, it will be important to continue the iterative process of message development with participants and young people to assure they are resonating and do not need to be more tailored by race/ethnicity as the program grows. In addition, having the navigator augment these messages with more personalized messaging based on the participants that are interacting with the program will also be crucial.

The use of media campaigns to change health-related behavior is not novel and has shown mixed results depending on the behavior being targeted and how long the impact lasts [40–43]. In particular, the type of desired behavior change is important to consider, with addictive behaviors notably harder to change when compared to behaviors that come with a level of enforcement, like seat belt wearing. While these campaigns are attempting to impact various health behaviors without additional programmatic support, our goal is to partner these messages with a navigator program that will ultimately provide continuous individual assistance to young people who desire contraception and assistance in overcoming barriers before, within, and after clinical encounters.

Our study has some limitations. First, we engaged adolescents in central Indiana and thus the messages may not resonate to all adolescents within the state. The sessions had diversity with prompts and scenarios to address the various geographic landscapes within our state, but it is possible that not all aspects were captured or illustrated in the final proposed messages. Second, the adolescents were asked to self-identify into only three distinct racial/ethnic groups and there exists many more groups within central Indiana. While analysis showed similar results in all three groups, it is possible that messages will not resonate with all adolescents. In addition, our inclusion criteria was limited to those that self-identified as females, leaving potential gaps in our messaging strategy for gender-diverse individuals. We also did not engage parents or sexual partners, who we know play an important role for many adolescents making decisions around contraception use. Finally, although the messages were refined through an iterative process, ongoing feedback will be necessary as this navigator program is piloted and grows.

### 4.2. Innovation

Human-centered design is not new, but its use within healthcare is still in its early stages when compared to other research methodologies. Engagement of ultimate end-users is necessary to create successful interventions. The combination of using HCD approaches to create an outreach strategy and navigator program to impact various elements of the reasoned action change model of behavior change is novel. Our findings are similar to other studies examining how to message reproductive health effectively with young people but augment the literature as they are focused on bringing young people outside of the healthcare system into contact with a navigator who can facilitate that.

Our study used an HCD approach which enabled messages to be created and edited with young people’s input and to identify key elements that are essential to be included. Our results illustrate the importance of having an outreach strategy that creates trust and provides individual focused information for young people interested in help obtaining contraception. These messages will be integral to the “IN Control” navigator program’s ultimate success. While this name has elements that are associated with the state of Indiana, choosing a program name that resonates for various reasons for the end users is important to keep in mind.

### 4.3. Conclusion

Our study involved adolescents as key stakeholders and HCD techniques in co-design of messages for a contraception navigator program named “In Control.” The goal of this program is to reach adolescents in Indiana seeking birth control by using a youth-informed messaging strategy and ultimately improve clinical care access within our state.

## Supplementary Material

1

2

3

4

## Figures and Tables

**Table 1:** Asynchronous human centered design sessions scenarios.

Scenario Prompt
Elle is 17 and thinking about having sex for the first time. She doesn’t know what to do about contraception. She is a good friend of yours and takes your thoughts and advice seriously. What would you say to encourage her to reach out to get support?
Bea is 16 and another friend of yours. She confided in you that she’s considering changing up her contraception game (her current pill is causing side effects), but she needs some help figuring out what will work for her. What would you say to encourage her to reach out for support in her area?
Carla is 15 and she is having sex without any protection. She knows this is a bad move, but she’s scared her parents will find out if she tries to get on birth control. What would you make to encourage her to reach out for support in getting contraception?

**Table 2:** Key insights from asynchronous data collection of communication model elements.

Illustrative Participant Statement	Insights for Prototype Messages	Opportunities and Program Characteristics
**Sender**		
*“I thought it was a good representation of someone getting the information that they need. It also might show Carla there are other people besides her parents that are there to support her.”*	Visual representation of recognizable people and organizations is important.	• Show who the navigator is and their association with the clinical providers
	Photographs of doctors and nurses were used in message prototypes as well as established medical organizations.	

**Receiver**		
*“Just take a survey, get connected to a person and create a plan to find the birth control for you!”*	Make it easy to get started	• Individual Focused • Acknowledge that they are smart and capable, but need help learning about and finding birth control
*“Struggling to find the perfect birth control? Take this quiz on Contraception Navigator to get started.”*		
*“Swipe to learn more.”*		
*“Getting birth control isn’t easy, and the process of finding one that works right can be intimidating at such a young age.”*		

**Message**		
*“This way, it helps to keep it simple and gives them the information and resources to use the program to get help finding the right contraception for them and how to obtain it.”*	Seeking birth control can be difficult	• Communicate that getting birth control can be easy • Navigator program makes learning about options easy
*“I made this because it shows her option of birth control. It shows her somewhere she can go without her parents if she needs to. As well as it shows that if your family loves you and you tell them everything will end out fine. As well as it shows that I love them.”*	Finding the right birth control can be different for each person	• The best birth control is the one that works for you. • There are some easy “wins” to getting on birth control. You can use condoms as you’re figuring out your birth control plan
*“I would make sure she is planning this with someone she trusts.”*		
*“I think the best option for her is to talk to someone with experience for example a doctor who specializes in reproductive health or someone who has had experience taking birth control.”*	Clients can benefit from having support throughout their birth control journey There are a lot of barriers to getting birth control.	• The program will help you even after you start using your birth control to make sure it’s a good fit for you. • Where are you getting hung up? We want to help you where you want help, not tell you what to do.
*“I thought it was a good representation of someone getting the information that they need. It also might show Carla there are other people besides her parents that are there to support her.”*	Teach clients how to talk about birth control comfortably and have a shared vocabulary with the client.	• Talking about birth control is okay, and you should feel comfortable talking about it. • Be in control of the conversation with your doctor - know your preferences and advocate for those. • Explain the words that are used and make sure they feel comfortable using those words too.
	Dispelling Myths about Birth Control	• Provide accurate and trusted information
*“Use our app to talk to certified medical professionals…”*	Explain what the navigator program does	• The navigator program is a safe space
*“So if you wanna talk about it or if you need any help, I’ll be right here.”*		• The navigator program is not pushy • The navigators are reliable and trustworthy

**Media**		
Participants used photographs of young women in their prototypes.	Represent your audience	• Use photos of diverse real people, the “everyday” teen
*“I wanted to make a list of different types of birth control. So I looked on planned parenthood’s website to find the most effective types. I made a list of what kinds they are, when they need to be taken/used, and their percentage of effectiveness.”*	Use visuals to provide information	• Show contraception options

**Environment**		
*“I think that advertising this program in platforms like Instagram will be very helpful for other people to reach out for information, especially younger people because social media is their main source of information.”*	Be visible and present where young people are	• Website as a source of information • Social Media platforms • Physical locations where young people will be

**Table 3:** Prototype messages.

There’s a lot to learn about birth control. We can help you learn about your options, so you feel comfortable with your decision.
The best birth control is the one that works for you, and you can pretty easily get and use condoms while you’re deciding on other forms of birth control.
The program will help you even after you start using birth control to make sure it’s a good fit for you.
It’s difficult to get birth control, we can help make it easier.
It’s okay to talk about birth control and we can help you feel comfortable talking about it.
This program is reliable and here for you. You can trust the information you get from us and know that we’re here to help you find your way, not push you in a certain direction.

**Table 4:** Message guidelines and final proposed messages.

Message Guidelines	Final Messages
Focused on the individual seeking birth control	“The best birth control is the one that works for you…that might be different than your friends/sisters/cousins and that’s okay.”
	“Your uterus is unlike any other! Treat it that way and find the birth control that works for you!”
Show the program is trustworthy	“Don’t settle for birth control rumors; we’ve got the trustworthy information you want.”
Feel open and inviting	“We’re not here to point you in a direction…we’re here to help you figure out which direction might work for you.”
Be short and to the point	“Figuring out the right birth control for you can be difficult…we can help.”
Explain how the program works	“You’re in the right place to get help getting the right birth control for you.”
Help the audience feel comfortable and learn about options	“Having a hard time figuring out which birth control you should use? We can help.”
	“There are a ton of birth control options out there, and a ton of people that will tell you which is best, but the best birth control is the one that works for you.”
Help the user feel protected	“We’ll keep checking in after you start using birth control to make sure it’s a good fit for you.”
Normalize talking about birth control	“It’s okay to talk about birth control and we can help you feel comfortable talking about it.”
Use citations to earn credibility and reliability	“We’ll give you information you can trust (including the source so you can look it up to be sure!).”
Acknowledge how difficult it can be for some people to figure out and obtain birth control	“We can help make it easier to get your hands on the birth control you want.”
