# Supplementary material for: A human-centered designed outreach strategy for a youth contraception navigator program
Source: PEC Innov. 2022 Oct 18;1:100093. doi: 10.1016/j.pecinn.2022.100093 (PMC9762731; doi:10.1016/j.pecinn.2022.100093)

Appendix A: Social Media Ad Copy and Images Used

“Looking for to make a positive impact? (and make a little spending money for your time) Click below to learn more about the study!”

“How does earning a little extra money and helping out with a really awesome cause sound? Click below to sign up!”

“How does helping out a great cause as well as making some extra cash sound 💸💸💸? We will just need your opinion and some of your time! 😀”

“Offer your opinion, help an amazing cause, and earn extra money, what could be better? Click below to learn more!”

“We are looking for someone to participate in our research program, just answer a few questions and offer your opinion!”

“We are looking for your opinion on access to birth control, offer your opinion and get paid!”

“Do you have an opinion on birth control access? Let us know your thoughts and make some extra cash!

Ad Images

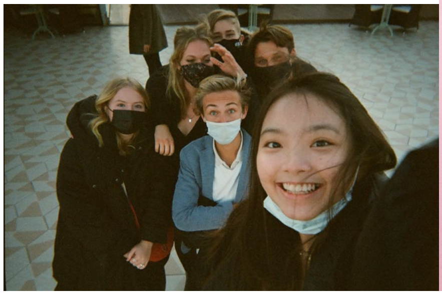
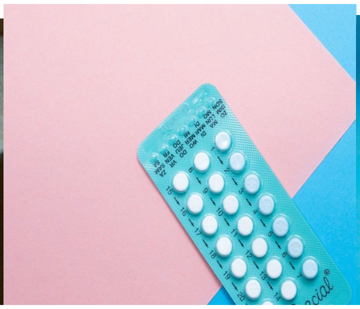
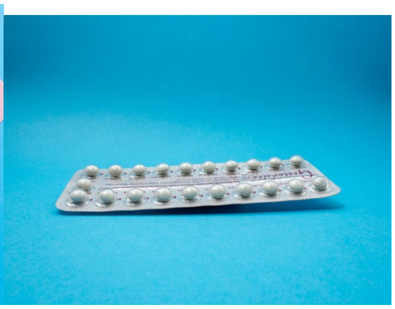
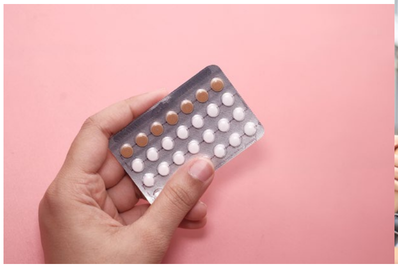
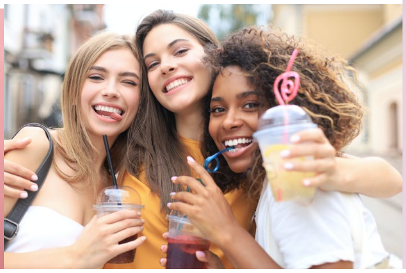
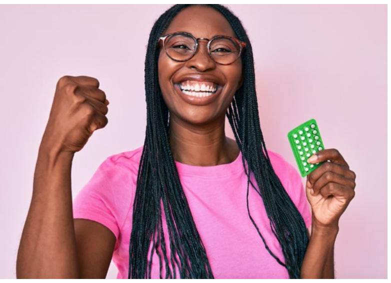

Supplement: Supplementary file 1 — Supplementary Appendix A [file mmc1.pdf]
