# Supplementary figures and images for: A human-centered designed outreach strategy for a youth contraception navigator program
Source: PEC Innov. 2022 Oct 18;1:100093. doi: 10.1016/j.pecinn.2022.100093 (PMC9762731; doi:10.1016/j.pecinn.2022.100093)

## Appendix C: Experience Mapping

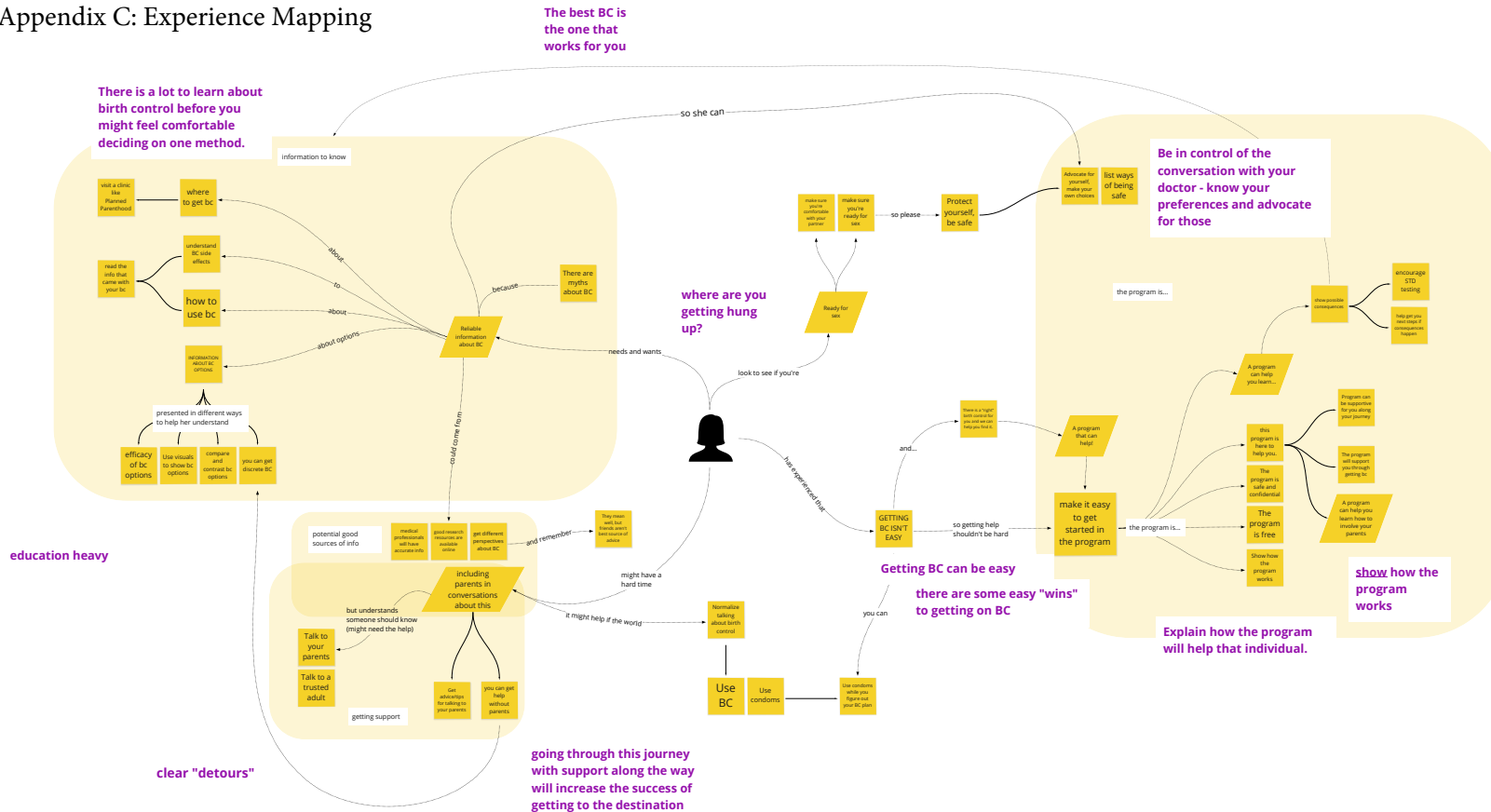

Supplement: Supplementary file 3 — Supplementary Appendix C [file mmc3.pdf]
