# Supplementary material for: A human-centered designed outreach strategy for a youth contraception navigator program
Source: PEC Innov. 2022 Oct 18;1:100093. doi: 10.1016/j.pecinn.2022.100093 (PMC9762731; doi:10.1016/j.pecinn.2022.100093)
Supplement: Supplementary file 4 — Supplementary Appendix D [file mmc4.pdf]

Appendix D: Personas developed during data analysis

Sa5m, H.S. junior

Don't listen to them, they've got an agenda

Sa5m is an activist and leader in her school. She's only started being more active with her boyfriend, and is firmly in the "everything, but" category of sexual activity. She sees how birth control is messaged differently for black girls and she distrusts the entire industry.

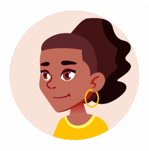

- afraid all information about BC is wrong
- negative past experiences with bc
- scared of long-term BC
- low access to medical care generally

JADE, 16

MOTIVATION FOR SEEKING BC
- Is getting pretty serious with her long-term boyfriend.
- Thinks she's ready to start having sex, but wants to be protected before her first time.
- She doesn't want to worry about getting pregnant.

SITUATION WITH PARENTS
- Her parents are no help at all -- they'd lose it if they knew; "sex before marriage is a sin."

KNOWLEDGE
- She's already done a lot of research, but there are too many options; just the number of different birth control pills is overwhelming.
- She's not sure how to get prescription birth control without her parents finding out; Can't they see it on the insurance bill?
- Thinks: "Maybe condoms are best because I can hide them and they're easy to get."
- Heard the IUD hurts REALLY bad to insert and has a lot of scary side effects.

LIFESTYLE
- Feels confident she can use any contraception as directed.

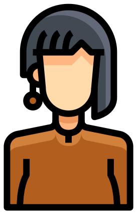

- parents will throw a shoe at them at the mention of a boy
- not yet having sex, but considering it
- sex-shaming background

Trisha, 17

Has used birth control in the past and had bad experience with it.

- Knows that she needs to be on birth control
- In a serious relationship with Tony
- Not sure she can afford birth control
- It's been difficult for her to get in to see her doctor in the past
- Past birth control messed with her weight and mood and her doctor didn't listen to her when she said she was having bad reactions.
- Doesn't know there are so many options (pills, shots, and condoms have been her past knowledge, was on pill before, now only uses condoms)

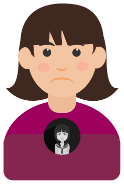

Trisha has been with her boy-toy Tony for a month now and it's super serious. They do it all the time and he always uses a condom (mostly...). In the past, Trisha was on the pill but it had some side effects she didn't like (weight gain and mood swings). When she was finally able to get in to talk to her doctor about it, he was dismissive about the side effects. So she decided to stop using it.
Trisha's ready to be on birth control again because she wants to make sure she's protected from unwanted pregnancy and heard how they're using condoms isn't always the most effective.

- low access to medical care generally
- in a serious relationship
- negative past experiences with bc

Torie, 15

on BC for non-sexual reasons. Period control ... control over my body

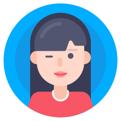

Torie has had just the worst period symptoms for years. She learned that she could get on a birth control that keeps her from having her period every month and wants to have that control over her body. She's not sure about long-term birth control options and has questions about those.
She's been taking the pill everyday, but her life is busy with school, running three clubs, and trying out for the cross-country running team next month. She's interested (albeit hesitant) to learn more about options for birth control that don't involve her taking something everyday.

- taking BC for non-sexual reasons
- scared of long-term BC
- busy life... doesn't know how does BC will fit into it

Skye, college freshman

I learned all this in high school. But now that I actually need it, I'm a little scared.

Skye doesn't need education she's in college. She already knows all this stuff. But no one is ever really talking about how it works in practice. Even condoms. She keeps hearing how people do it wrong, but no one says how. So is she doing it wrong? You just roll it down, and then squeeze the tip or something right? Also, she's on some anti anxiety meds, and no one ever said if that will be affected.

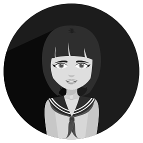

- "education" sites turn her off.
- safe sex information in practice is underrepresented
- don't talk down to her
- her situation is unique (anti anxiety, school stress)

- scared of taking hormones
- knows about bc options but needs a sounding board
- Knows everything... except which one works for her
- influencer: the girl at school that all the other girls get tips from
