# Supplementary material for: A human-centered designed outreach strategy for a youth contraception navigator program
Source: PEC Innov. 2022 Oct 18;1:100093. doi: 10.1016/j.pecinn.2022.100093 (PMC9762731; doi:10.1016/j.pecinn.2022.100093)

Appendix B: Affinity Diagramming. Each colored square on this board represents one theme from the three separate design sessions affinity diagrams that were combined to examine the overlap between the populations.

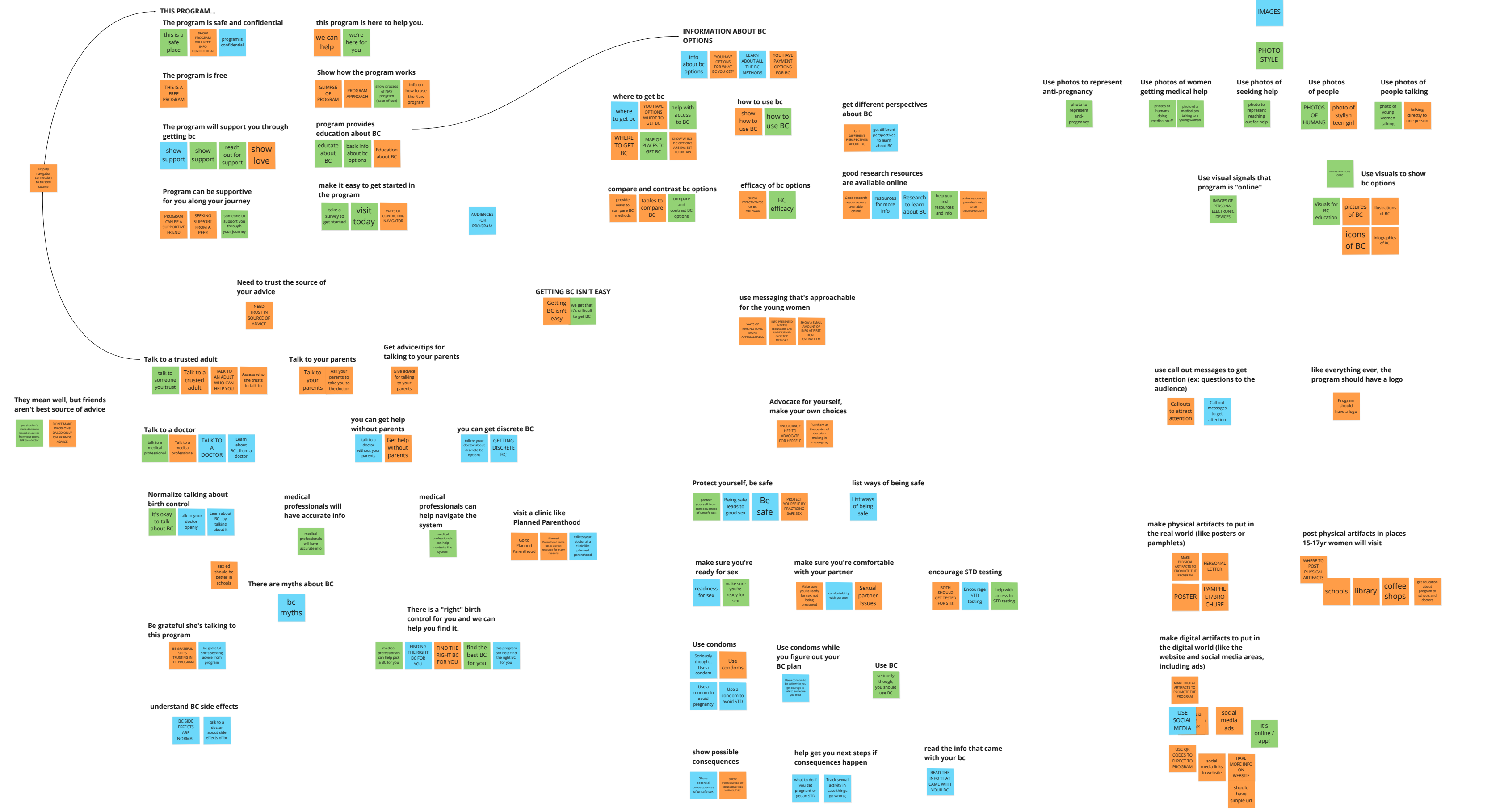

Supplement: Supplementary file 2 — Supplementary Appendix B [file mmc2.pdf]
